# Supplementary material for: Orbitronics: light-induced orbital currents in Ni studied by terahertz emission experiments
Source: Nat Commun. 2024 Mar 6;15:2043. doi: 10.1038/s41467-024-46405-6 (PMC10917802; doi:10.1038/s41467-024-46405-6)
Supplement: Supplementary file 1 — Supplementary Information [file 41467_2024_46405_MOESM1_ESM.docx]

**Supplementary Information**

**1. Properties of hot-electron orbital transport in Ni/Cu/MgO**

Figure S1 Sketch of orbital transport in Ni/Cu/MgO structures. An out-of-equilibrium orbital momentum density *μ_L_* (accumulation) is generated from Ni by an ultrafast laser pulse, then propagates through Cu in a ballistic way up to the Cu/MgO. The different propagation ballistic paths are labeled by the cone aperture *θ_k_.*

We are searching for the time *t* and 1-dimensional space profile ($z$) of the orbital accumulation $\mu_{L}(z,t)$ in the pure ballistic regime of injection for the calculation of the Inverse Orbital Rashba effect (ORE).

Following the work of Tom Seifert et al.^1^, we deduce:

$\mu_{L}\left( z,t \right)=\frac{n_{L}}{S} \int_{0}^{1} d\left( \cos\theta\right) \delta\left( z-v_{o}t \cos\theta\right)\ldots[1]$with $n_{L}$ the number of out-of-equilibrium orbital carriers, *S* the surface of the pump laser spot, $v_{0}$ the average velocity of orbital carriers. $\frac{n_{L}}{S}$ represents the 2-dimensional orbital density injected at the Ni/Cu interface at the origin time t=0.

The equation [1] is different from the results of Seifert et al. (Eqn.[6-9] in their article) in that the latter considers the orbital-current density instead of the orbital density itself.

Once we consider a certain probability of an orbital flip $p_{of}=r_{of}\tau_{D}$scaled by a certain orbital-flip rate $r_{of}=\frac{1}{\tau_{of}}$ (typically by orbital decoherence) with $\tau_{D}=d_{Cu}/v_{o}$ the typical delay time between injection into the Cu layer from Ni and detection at the Cu/MgO interface, one can express $\mu_{L}\left( z,t \right)$ according to:

$\mu_{L}\left( z,t \right)=\frac{n_{L}}{Sv_{o}t} \int_{0}^{1} d\left( \cos\theta\right) exp\left( -\frac{t}{\tau_{of}} \right)\delta\left( \frac{z}{v_{o}t}- \cos\theta\right)$ thus giving:

$\mu_{L}\left( z,t \right)=\frac{n_{L}}{S} \frac{1}{v_{o}t}exp\left( -\frac{t}{\tau_{of}} \right)\Theta\left( z-v_{F}t \right)\ldots[2]$with $\Theta$ the Heaviside function expressing thus the orbital accumulation (orbital momentum density) $\mu_{L}\left( z,t \right)$ vs. the coordinate z in the ballistic regime of orbital injection generated by a $\delta$- Dirac optical pulse $\delta(z)\delta(t)$ at the Ni/Cu interface (z=0).

The calculation of the inverse Orbital Rashba effect (IORE) has been performed by considering a ballistic trajectory of the orbital moment accumulation in the three directions crossing the Cu interlayer from the Ni /Cu interface and possibly subject to orbital flip event at a rate $r_{of}=\frac{1}{t_{of}}.$ The velocity distribution has been considered as isotropic *vs.* angle at z=0 (interface Ni/Cu) with an equal distribution of the orbital momentum density.

We will now consider the experimental situation of a short optical pulse R(t) of temporal width Δτ generated at z=0. We can model the shape of the pulse according to:

$R\left( z,t \right)=\frac{1}{\sqrt{\pi}\Delta}exp\left( -\frac{t^{2}}{\Delta^{2}} \right)\delta(z)$normalized to a unity power. The subsequent expression for $\mu_{L}\left( z,t \right)$ is then generalized into:

$\mu_{L}\left( z,t \right)=\frac{n_{L}}{S} \int_{t_{o}}^{\infty} \frac{d\tau}{\tau}exp\left( -\frac{\tau}{\tau_{of}} \right)R\left( t-\tau\right)\ldots\left[ 3 \right]$as obtained from a convolution product with $R\left( z,t \right)$. Here, $t_{0}=\frac{d_{Cu}}{v_{o}}$ is defined as the minimum carrier flying time through the Cu layer corresponding to an exact normal incidence ($\cos\theta=1$).

**2. THz Traces:**

In the dipolar emission process, the shape of the THz electric field $E\left( t \right)$ generated by IORE follows:

$${E\left( t \right) \propto\dot{\mu}}_{L}\left( z,t \right)=\frac{n_{L}}{S} \int_{t_{0}}^{\infty} \frac{d\tau}{\tau}exp\left( -\frac{\tau}{\tau_{of}} \right)\dot{R}\left( t-\tau\right)\ldots\left[ 4 \right]$$

displaying two consecutive extrema (a minimum and a maximum). The respective minimum/maximum of the E-field $E\left( t \right)$ vs. the time t correspond to:
${\dot{E}\left( t \right) \propto\ddot{\mu}}_{L}\left( z,t \right)=\frac{n_{L}}{S} \int_{t_{0}}^{\infty} \frac{d\tau}{\tau}exp\left( -\frac{\tau}{\tau_{of}} \right)\ddot{R}\left( t-\tau\right)=0\ldots[5]$


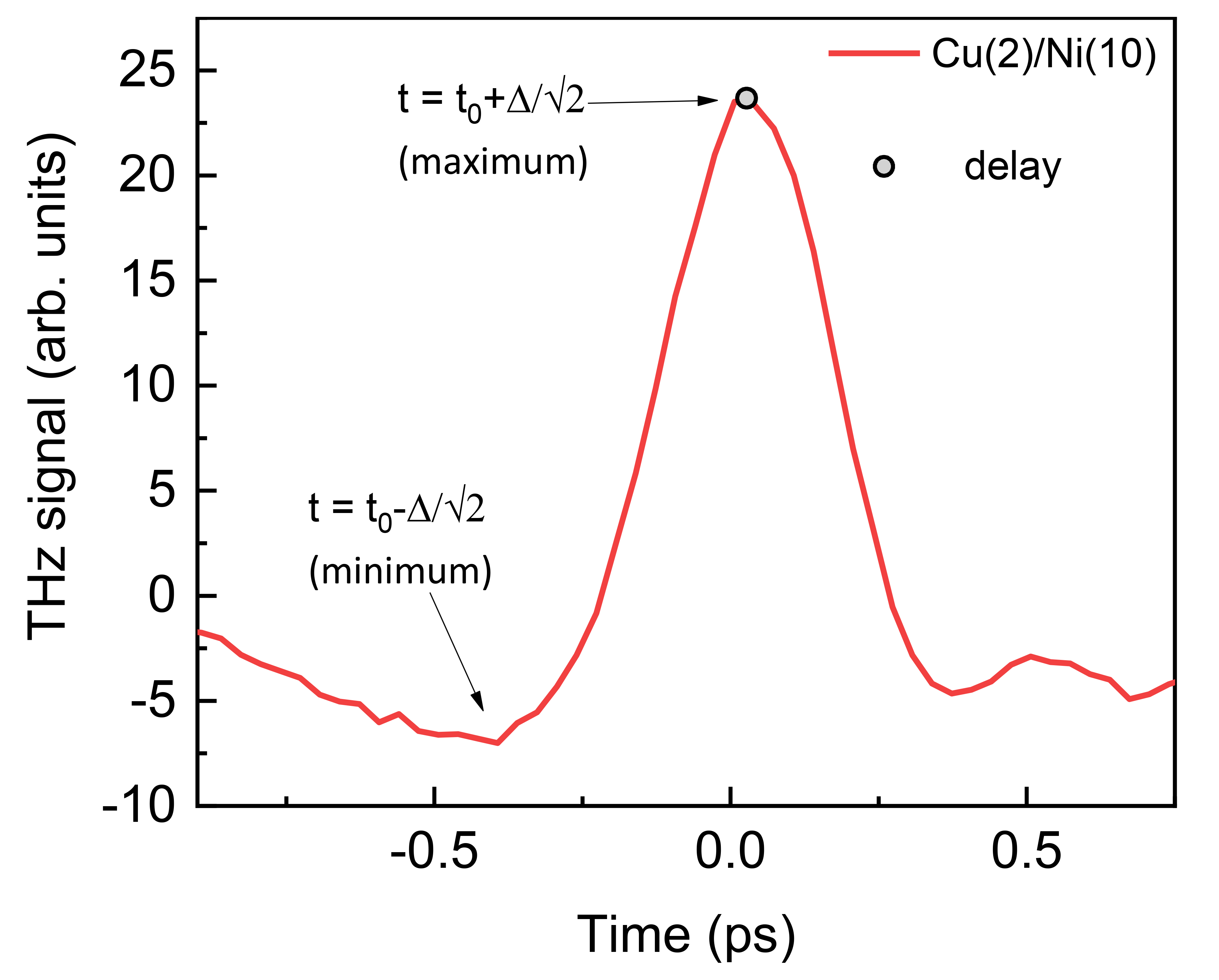


Figure S2 The respective time corresponding to the two extrema.

***2.1 Rough estimation:***

In the case of a rapidly varying function $R\left( t \right)$, that is varying in a timescale shorter than $\tau_{of}$ and $t_{0}$($\Delta{"t}_{0}, \tau_{of}$), one has $\ddot{R}\left( t-\tau\right)\approx0$ corresponding to an extremum of the THz trace thus leading to:

$\ddot{R}\left( t-\tau\right)\sim\left( \frac{4 \left( t-t_{0} \right)^{2}}{\Delta^{4}}-\frac{2}{\Delta^{2}} \right) exp\left( -\frac{t^{2}}{\Delta^{2}} \right)=0$giving the trivial condition $t=t_{0}\pm\frac{\Delta}{\sqrt{2}}$ (Fig. 1) as well as the linear relationship linking the delay time to the group velocity as: $\tau_{D}=\frac{d_{Cu}}{v_{o}}$ as observed in Ref. 1. Such simple linear relationship is valid in the limit of a very short orbital relaxation time unlike our present case.

***2.2 Accurate calculations at the order beyond:***

For longer orbital relaxation time like observed e. g. in Cu, one must go beyond the leading order.

By writing $\ddot{R}_{\left( t-\tau\right)}=\ddot{R}_{\left( t-t_{o} \right)}$ - $R_{\left( t-t_{o} \right)}\left( t-t_{o} \right)$ and inserting in the integral [5], one obtains the self-consistent equation giving the position of the minimum/maximum of the E-field:

$\ddot{R}_{\left( t-t_{o} \right)}=\left( -t_{o}+\frac{\Lambda^{(0)}}{\Lambda^{(-1)}} \right) R_{\left( t-t_{o} \right)}$with the definition of the general $\Lambda^{(n)}=\int_{t_{o}}^{\infty} d\tau\tau^{n} exp\left( -\frac{}{\tau_{of}} \right)$ functions.

We arrive at $\left( 2\left( \frac{t-t_{o}}{\Delta} \right)^{2}-1 \right)=\left( -t_{o}+\frac{\Lambda^{(0)}}{\Lambda^{(-1)}} \right)\left( 6\frac{\left( t-t_{o} \right)}{\Delta^{2}}-4\frac{\left( t-t_{o} \right)^{3}}{\Delta^{4}} \right)$ allowing the determination of $t=t\left( t_{o},\Delta\right)$. At the first order of development, one finally finds:

$t=\frac{\Lambda^{(0)}}{\Lambda^{(-1)}}\pm\frac{\Delta}{\sqrt{2}}\ldots\left[ 6 \right]$together with the time delay of the THz spectra compared to a reference point of thickness $dmin$:

$\tau_{D}(d)=\frac{\Lambda^{(0)}}{\Lambda^{(-1)}}\left( d \right)-\frac{\Lambda^{(0)}}{\Lambda^{(-1)}}\left( dmin \right)$ with $\frac{\Lambda^{(0)}}{\Lambda^{(-1)}}\left( d \right)=\frac{\int_{t_{o}}^{\infty} dt exp\left( -\frac{t}{\tau_{of}} \right)}{\int_{t_{o}}^{\infty} dt \frac{1}{t} exp\left( -\frac{t}{\tau_{of}} \right)}.$

From our THz experiments in the time domain, the following equation is used for fitting.

$$\tau_{D}=\frac{\int_{t_{o}}^{\infty} dt exp\left( -\frac{t}{\tau_{of}} \right)}{\int_{t_{o}}^{\infty} \frac{dt}{t}exp\left( -\frac{t}{\tau_{of}} \right)}$$

We then obtain typical ballistic values for the respective group velocity and time: $v_{o}$=0.26 nm/fs and $\tau_{of}$=350 fs (Figure 2e). From this information, the expected orbital diffusion length $l_{of}$ in the diffusive regime of transport may be evaluated to:$l_{of}= v_{o}\sqrt{\tau_{of}t_{p}/3}$, where $t_{p}$ is the typical scattering momentum relaxation time (typically 10 fs for Cu at Room temperature). Therefore, the orbital diffusion length in Cu may be estimated to be 9 nm.

**3. Influences of the polarization of the pump laser**

Following the method used by Huisman et al^2^, we pump the samples with femtosecond laser of $\sigma^{+},\sigma^{-}$, and linear helicities, and recorded the terahertz emission. Figures S3 a and b show time traces of the x component (a: perpendicular to the magnetic field) and the y component (b: parallel to the magnetic field) of the terahertz emission for $\sigma^{+},\sigma^{-}$, and linear helicities. We observe that both the x component and the y component remain the same, regardless of the laser helicity.


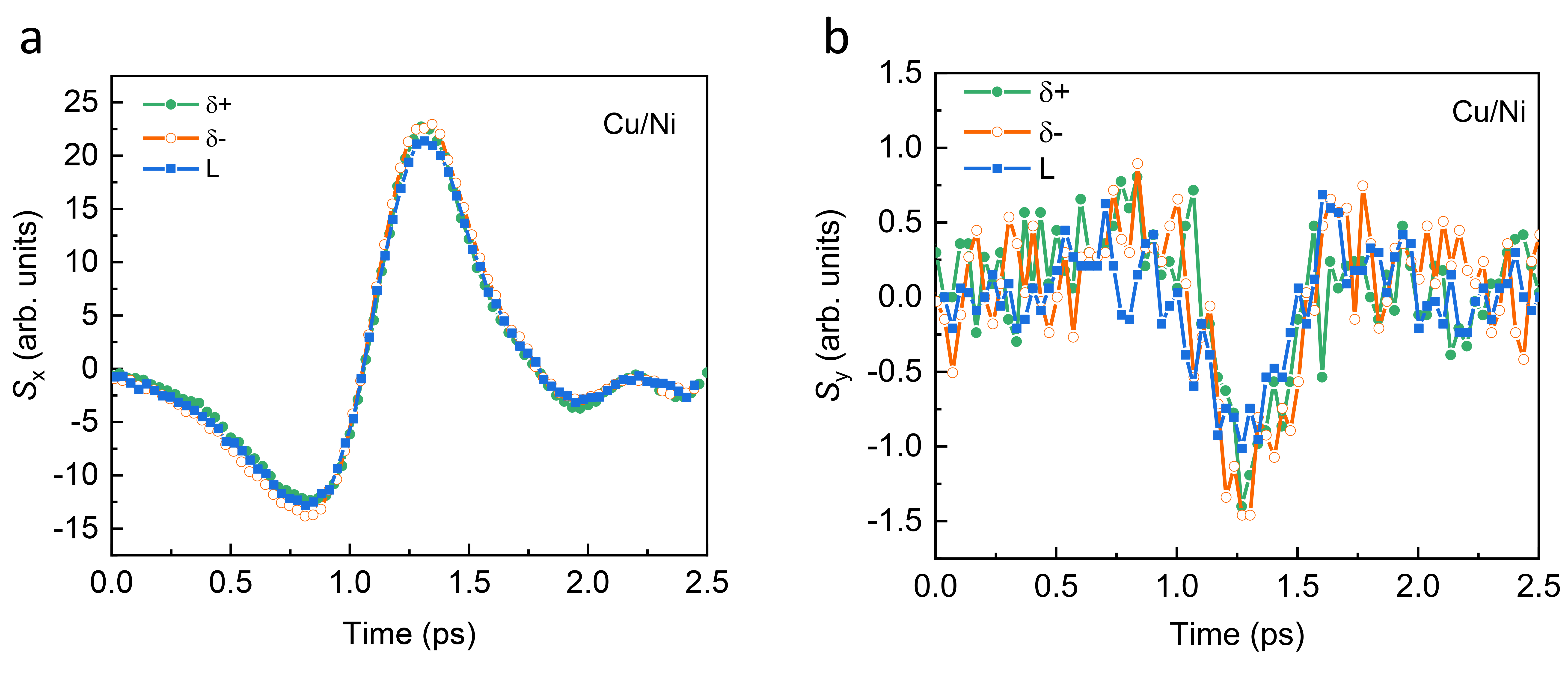


Figure S3 The x component (a: perpendicular to the magnetic field) and the y component (b: parallel to the magnetic field) of the terahertz emission induced by pump lasers of $\sigma^{+},\sigma^{-}$, and linear helicities.

**4. Study of THz emission from** **MgO/Cu/Ni(2) samples**

We studied a series of Ni single-layer samples to study the thickness dependence of THz emission in MgO/Cu/Ni (2 nm) samples. Only AHE contributions are expected for single layers, and the extremely small THz emission seen for a 2 nm single layer of Ni shows that the AHE contribution is negligible in this very thin limit, in agreement with the sharp drop of the AHE contribution to THz emission at decreasing thickness in other ferromagnets ^3^. Therefore, the AHE contribution can be neglected in MgO/Cu/Ni (2 nm), and the THz emission is ascribed to only orbital contributions, as discussed in the text. Figure S4a shows the waveforms from MgO/Cu/Ni (2 nm) samples. The extracted peak amplitude is shown in Figure 2c (red curve).


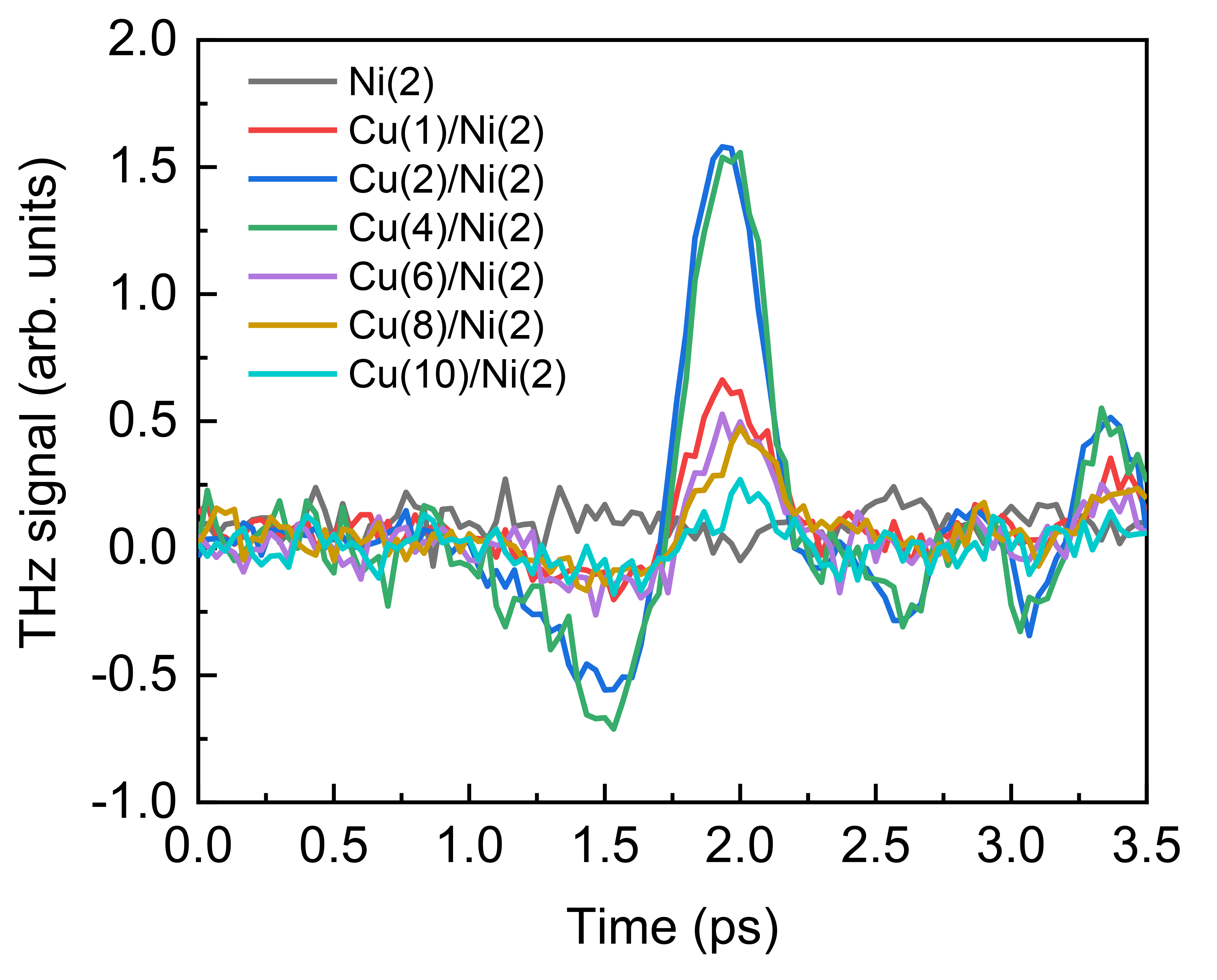


Figure S4 THz waveforms for substrate//MgO/Cu/Ni (2 nm) samples for different Cu thicknesses. The extracted peak amplitude is shown in Figure 2c (red curve).

**5. Study of THz emission from MgO/Cu/CoFeB****(10 nm)**

Figure S5 shows the THz emission from MgO/Cu/CoFeB(10 nm). The terahertz emission from Cu/CoFeB samples remains roughly constant for the Cu thickness less than 6 nm (Figure S5). Terahertz emission from MgO/Cu/CoFeB is mainly driven by AHE. This means that, at least for CoFeB, adding Cu layers to a magnetic layer in this thickness range, does not change significantly the AHE contribution to THz emission. This result cannot be reliably extended to Ni, except for very thin layers for which the AHE contributions to emission becomes extremely small^3^ and can be neglected.


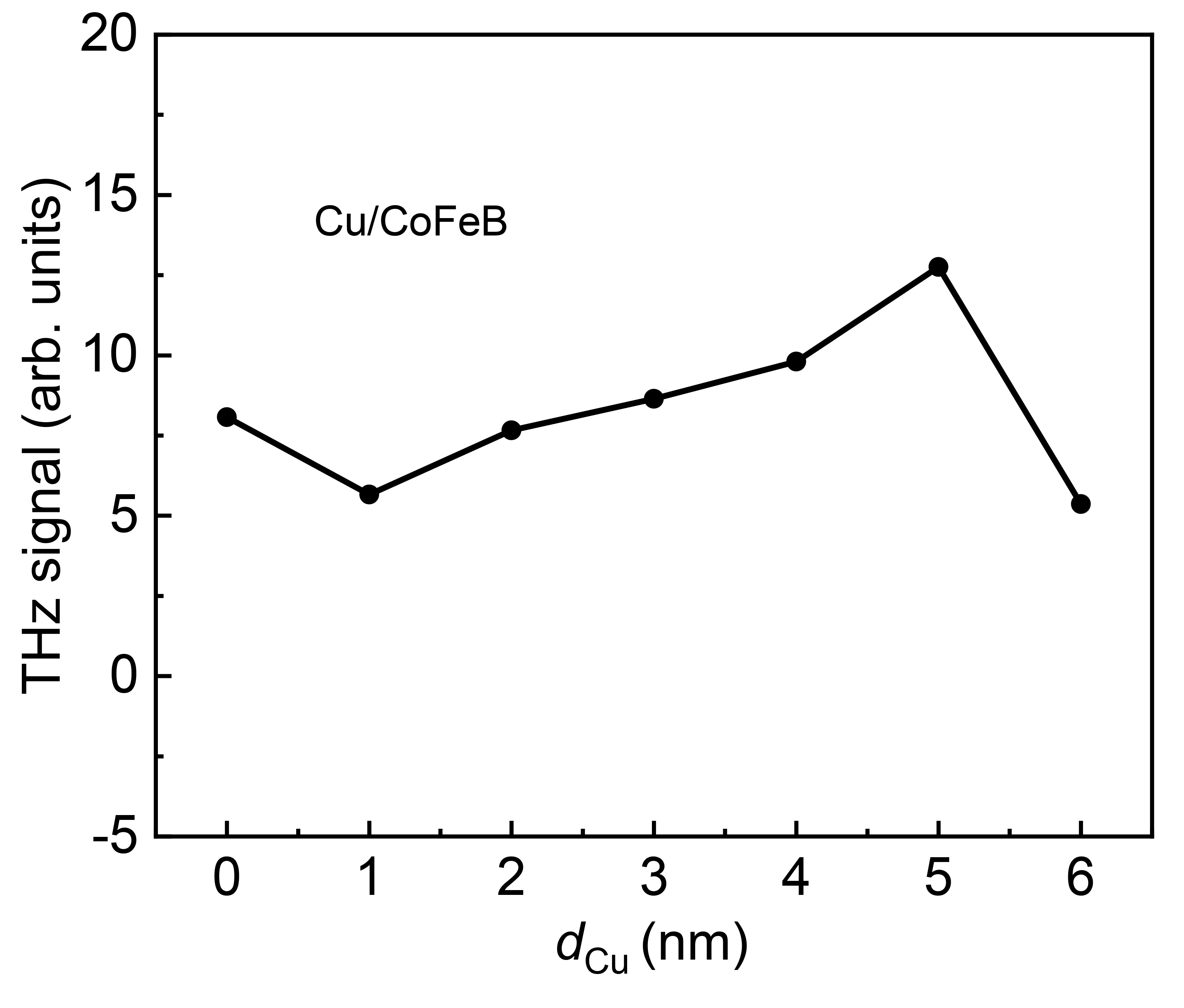


Figure S5 THz peak amplitudes from raw data of Cu/Ni (10 nm) as a function of the Cu thickness.

**6. FWMH and FFT study of the THz waveforms from Cu/Ni (10 nm) heterostructures**

We extracted FWHM and performed FFT on all the data in Fig. 2d. The FWHMs and FFT spectra (Fig. S6) are the same within the error range, showing that there is no increase in the width of the waveform.


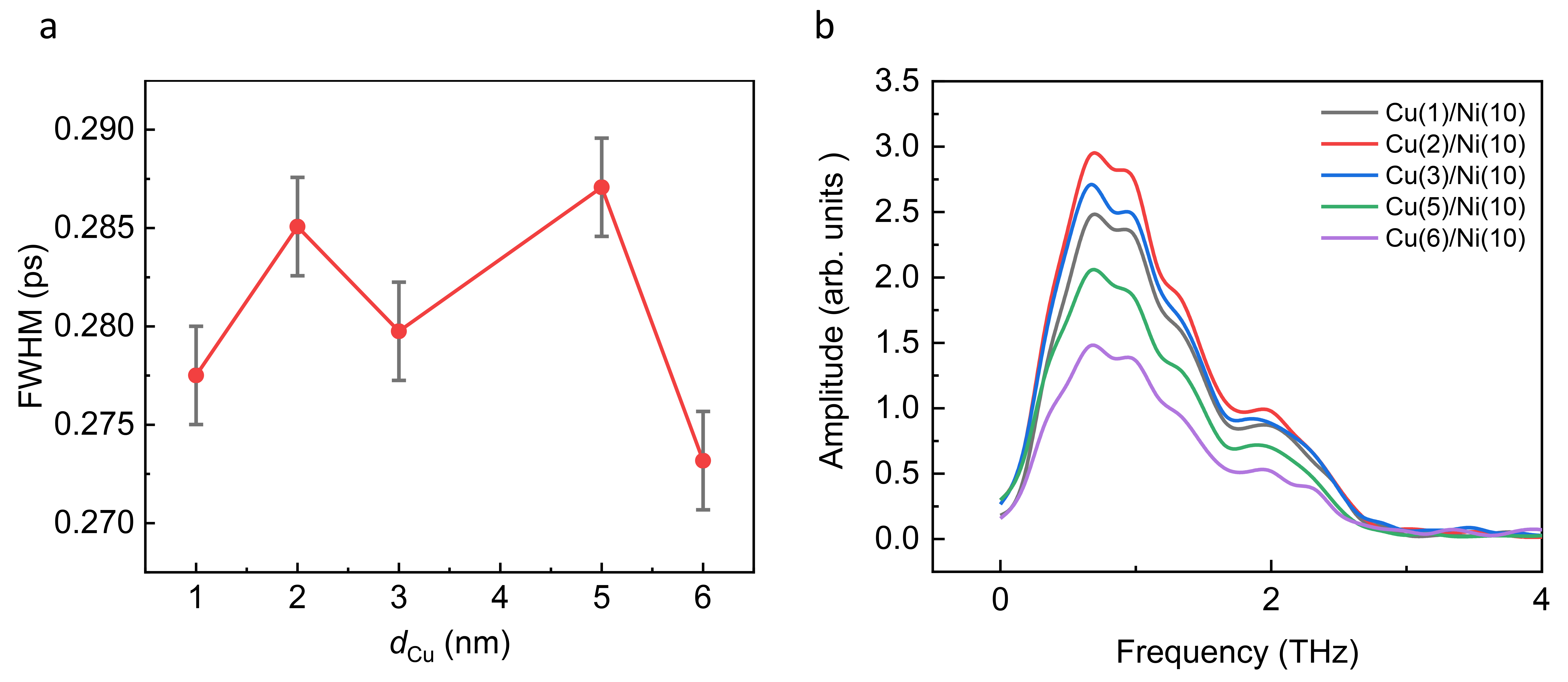


Figure S6 (a) FHWMs and (b) Fourier spectra of the THz waveforms from Cu/Ni (10 nm) heterostructures with varying Cu thickness. The errorbar (vertical line at the datapoints) is estimated based on the multiple scans of a given sample.

**7. Reference waveforms without timeshift**

Figure S7 shows the experimental results obtained on non-orbital-Rashba systems CoFeB/Ta (bulk spin-ISHE) and used as a “reference system” free of any interfacial orbital Rashba effect that does not show any timeshift with the same experimental protocol on the same optical experimental bench.


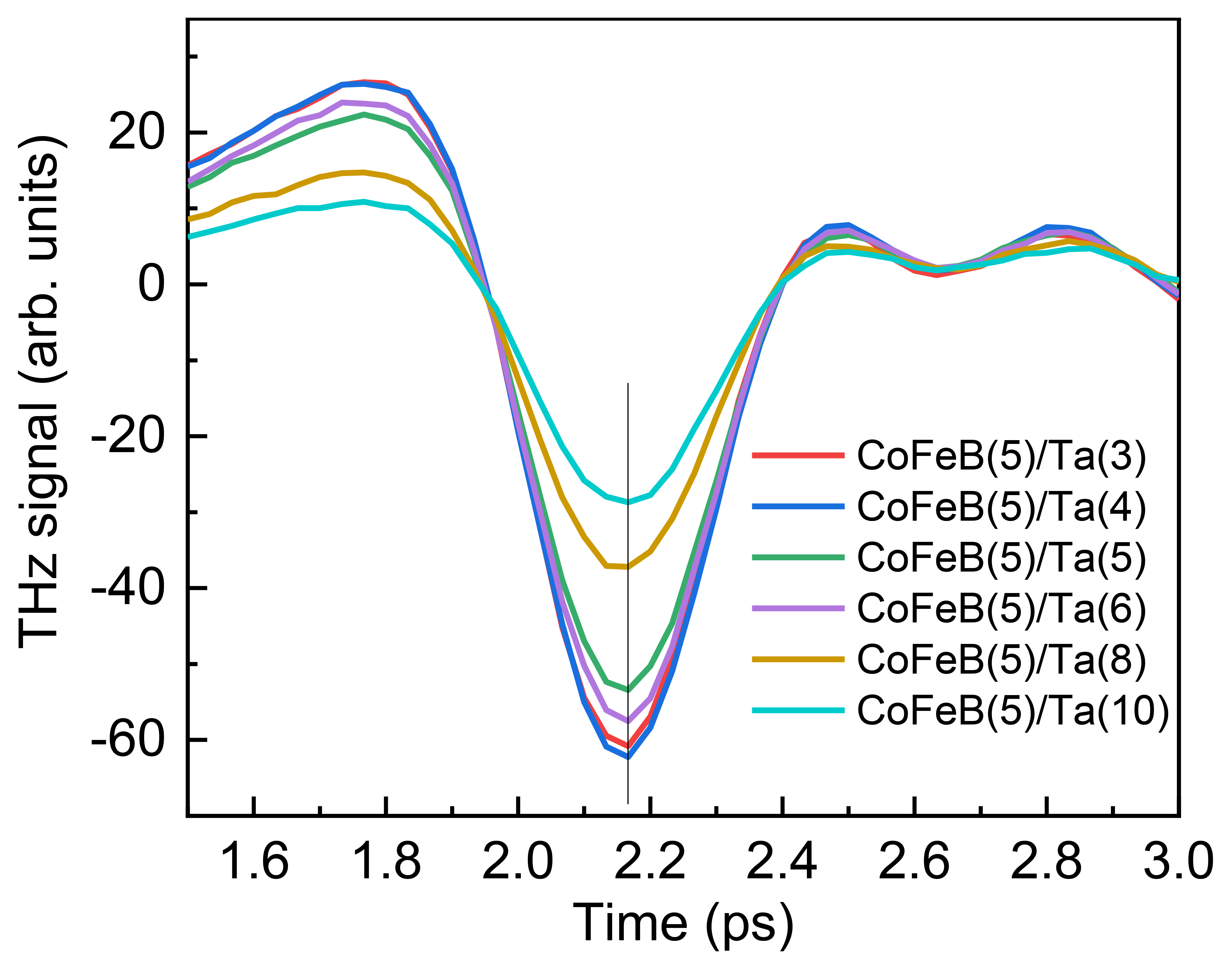


Figure S7 Experimental results obtained on non-orbital-Rashba systems.

**References**

1. Seifert, T. S. *et al.* Time-domain observation of ballistic orbital-angular-momentum currents with giant relaxation length in tungsten. *Nat. Nanotechnol.* 1–7 (2023) doi:10.1038/s41565-023-01470-8.

2. Huisman, T. J. *et al.* Femtosecond control of electric currents in metallic ferromagnetic heterostructures. *Nat. Nanotechnol.* **11**, 455–458 (2016).

3. Mottamchetty, V. *et al.* Direct evidence of terahertz emission arising from anomalous Hall effect. *Sci. Rep.* **13**, 5988 (2023).
